# Shiga Toxins Produced by Enterohaemorrhagic *Escherichia coli* Induce Inflammation in Toxin-Sensitive Cells through the p38 MAPK/MK2/Tristetraprolin Signaling Pathway

**DOI:** 10.4014/jmb.2410.10016

**Published:** 2024-11-22

**Authors:** Seo Young Park, Yu-Jin Jeong, Kyung-Soo Lee, Jun-Young Park, Jongsun Park, Vernon L. Tesh, Moo-Seung Lee

**Affiliations:** 1 Environmental Diseases Research Center, Korea Research Institute of Bioscience and Biotechnology, Daejeon 34141, Republic of Korea; 2 Department of Biomolecular Science, KRIBB School of Bioscience, Korea University of Science and Technology (UST), Daejeon 34113, Republic of Korea; 3 Department of Pharmacology, College of Medicine, Chungnam National University, Daejeon 35015, Republic of Korea; 4 Department of Medical Science, Metabolic Syndrome and Cell Signaling Laboratory, Institute for Cancer Research, College of Medicine, Chungnam National University, Daejeon 35015, Republic of Korea; 5 Department of Microbial Pathogenesis and Immunology, Texas A&M University, College of Medicine, Bryan, TX 77807, USA

**Keywords:** EHEC, Shiga toxins, bacterial toxins, inflammation, HUS, food-borne pathogen

## Abstract

Shiga toxins (Stxs), produced by *Shigella dysenteriae* serotype 1 and certain *Escherichia coli* pathotypes, cause hemorrhagic colitis, which can progress to hemolytic uremic syndrome (HUS) and central nervous system (CNS) pathology. The underlying mechanisms of toxin-induced inflammation remain unclear. The p38 mitogen-activated protein kinase (MAPK) and its downstream target, MAPKAPK2 (MK2), play key roles in various cellular responses. We identified Tristetraprolin (TTP) as a novel substrate of MK2 in Stx-intoxicated cells. Western blot analysis showed that Stxs induce phosphorylation of MK2 (Thr334) and TTP in globotriaosylceramide (Gb_3_)-positive cells, including D-THP-1 macrophage-like cells and HK-2 renal epithelial cells, but not in Gb_3_-negative T84 colon carcinoma cells. After treatment with wild-type Stx, the activity of phosphorylated MK2 and TTP persists for up to 8 h, while Stx2a^mut^, which lacks N-glycosidase activity, causes transient MK2/TTP phosphorylation. This suggests that Stxs selectively mediate MK2 and TTP activation in a Gb_3_-dependent manner. Knockdown of TTP in Stx2a-treated D-THP-1 cells upregulates proinflammatory cytokines such as TNF-α, IL-1β, IL-6, IL-8, MCP-1, and MIP-1α. The MK2 inhibitor PF-3644022 significantly reduces TTP phosphorylation and blocks the production of IL-6, IL-8, MCP-1, and MIP-1α in Stx2a-stimulated HK-2 cells. In conclusion, the MK2-TTP signaling pathway regulates the inflammatory response induced by Stxs in toxin-sensitive cells.

## Introduction

Shiga toxin-producing *E. coli* (STEC) strains, similar to *S. dysenteriae* serotype 1, pose an increasing threat to public health. Infections by STEC lead to hemorrhagic colitis, which can often progress into hemolytic uremic syndrome (HUS), a severe complication characterized by thrombocytopenia, anemia, and acute kidney failure [1, 2]. The frequency of STEC infections worldwide is estimated to be around 43.1 acute illnesses per 100,000 people per year, leading to 3,890 annual cases of STEC-HUS. Despite the much lower incidence of STEC-HUS in adults compared to children, most STEC-HUS-related deaths occur in people ≥60 years old. Treatment of STEC infection is very challenging because the use of certain antibiotics (β-lactams, fluoroquinolones, and cotrimoxazole) and antimotility agents during hemorrhagic colitis increases the risk of developing HUS [3-6]. STEC infections are not limited to humans, with some strains causing piglet edema disease, leading to losses in pig production [7]. Patients may be infected by contaminated food and water intake, person-to-person transmission, or contact with animal carriers of STEC [8].

Stx toxins are broadly divided into two types: Shiga toxin type 1 (Stx1) and Shiga toxin type 2 (Stx2). Stx1 toxins are divided into four subtypes, Stx1a, c, d, and e, whereas Stx2 toxins are divided into 12 subtypes, Stx2a through to Stx2l [10-12]. STEC are known to produce two Stxs: Stx1a, which is identical to the toxin secreted by *S. dysenteriae* serotype 1, and the genetically distinct Stx2a. Stxs display an AB5 structure with one catalytic ‘A’ subunit and a pentamer of ‘B’ subunits, which predominantly interact with globotriaosylceramide (Gb_3_), and to a lesser extent with globotetraosylceramide (Gb_4_) and globopentaosylceramide (Gb_5_) [2, 13].

After binding of Stxs to host receptors, the toxin is internalized and translocated first to the *trans*-Golgi network, Golgi apparatus, and then to the endoplasmic reticulum (ER), where they release the catalytically active A_1_ fragment (27 kDa) of the full A subunit (A_1_+A_2_) (32 kDa) into the cytosol [14]. There, the catalytic A domain fragment removes a single adenine residue from 28S rRNA (A4324 in rats) in the 60S ribosomal subunit, thus activating a signaling cascade that directly inhibits protein synthesis, inducing multiple host cell responses including inflammation and apoptosis [2, 15]. In the host GI tract, Stxs are transported across the intestinal epithelium by M cells. Stxs activate the immune response in Peyer’s patches containing innate immune cells such as macrophages, monocytes, and neutrophils. It has been well characterized that the Stxs-induced proinflammatory response facilitates organ damage, such as disruption of the intestinal microvasculature and renal tubular epithelium, by triggering dysregulated cytokine and chemokine production [16]. Numerous studies have characterized the p38 mitogen-activated protein kinase (MAPK) and its downstream target Mitogen-activated protein kinase-activated protein kinase 2 (MAPKAPK2 or MK2) signaling pathway in a variety of cell types. Stxs induce various stress-activated kinase pathways, including p38 MAPK, JNK, ERK, and MK2, which increase the synthesis of proinflammatory cytokines [17, 18].

MK2 phosphorylated by p38 MAPK can subsequently phosphorylate heat shock protein 25 (Hsp25), Hsp27, and Tristetraprolin (TTP) [19]. TTP is an RNA-binding protein reported to play a key role in inflammatory responses.

TTP binds to Adenylate-uridylate-rich elements (AREs) in 3'-untranslated regions (3'-UTRs) of target mRNAs, promoting their destabilization [20]. Potential approaches for targeting this TTP mRNA to treat inflammatory diseases include blocking the proteins that promote its binding and degradation using specific types of antisense oligonucleotides, such as 'stereo-blocking oligonucleotides'. Additionally, cell-based screening programs could be employed to elucidate the mechanisms regulating TTP expression and related proteins. Promoting TTP dephosphorylation is also a promising approach for managing chronic inflammatory diseases [21]. While LPS-treated wild-type (WT) bone marrow-derived macrophages (BMDMs) increased expression of TNF-α, an MK2 knockout, MK2/3 double knockout, and TTP-S52A-S178A knockin BMDMs showed decreased expression of TNF-α [22, 23]. Activation of TTP by p38 MAPK-MK2 can trigger the decay of cytokine mRNA. There is, however, a paucity of data on the mechanism by which Stxs induce MK2-TTP signaling in host cells.

Therefore, we investigated whether Stxs are involved in MK2/TTP-dependent inflammatory cytokine production. We show that Stxs induce phosphorylation of MK2 at threonine (Thr) 334, in addition to TTP phosphorylation, which precedes inflammatory cytokine production in toxin receptor Gb_3_-positive macrophage-like differentiated THP-1 (D-THP-1) and HK-2 human proximal tubule epithelial cells, but not in Gb_3_-negative T84 human colon carcinoma cells using western blot, enzyme-linked immunosorbent assay (ELISA), and Quantitative real-time PCR (qRT-PCR).

## Materials and Methods

### Toxins

Purified Stx2a, Stx2-B subunit (Stx2B) and Stx2a^mut^, harboring the triple mutation Y77S/E167Q/R170L in the enzymatic active site of the A subunit, were purchased from NIAID (NIH Biodefense and Emerging Infections Research Repository; BEI Resources, USA). Stx1a was a kind gift from Prof. Vernon L. Tesh at Texas A&M University, Texas, US. In the Vernon L. Tesh lab, Stx1a was purified from recombinant Stx1a-expressing *E. coli* DH5α (pCKS112) by sequential ion exchange and immunoaffinity chromatography. The purity of Stx1a was determined by sodium dodecyl sulfate-polyacrylamide gel electrophoresis (SDS-PAGE), silver staining, and western blot analysis. The level of endotoxin contaminants was reduced to <0.1 ng/ml using ActiClean Etox columns (Sterogene Bioseparations, USA). The degree of endotoxin contamination was assessed by a *Limulus* Amoebocyte lysate assay (Associates of Cape Cod, East USA).

### Antibodies

Rabbit monoclonal antibodies specific for human phospho-MAPKAPK-2 (Thr334), phospho-MAPKAPK-2 (Thr222), Tristetraprolin, and MAPKAPK-2, in addition to an HRP-conjugated monoclonal antibody specific for human β-actin and secondary antibodies specific for mouse or rabbit IgG, were purchased from Cell Signaling Technology (USA). SB203580 and PF-3644022 were purchased from Sigma-Aldrich, Inc. (USA).

### Cell Culture

The human myelogenous leukemia cell line THP-1 (American Type Culture Collection, USA) was cultured in RPMI 1640 medium (Corning, Thermo Fisher Scientific, USA) containing 10% fetal bovine serum (FBS; Gibco, Thermo Fisher Scientific), 5.0 μg/ml streptomycin, and 5.0 U/ml penicillin at 37°C with 5% CO_2_ in a humidified incubator. THP-1 cells (1.0 × 10^6^ cells/well) were seeded in 12-well plates and differentiated to an adherent macrophage-like state with phorbol 12-myristate 13-acetate (PMA; Sigma Chemical Co., USA) at a concentration of 50 ng/ml for 48 h. The differentiated THP-1 cells were washed three times with cold, sterile Dulbecco’s phosphate-buffered saline (DPBS; Sigma Chemical Co.) and then incubated with fresh complete medium lacking PMA. The medium was changed every 24 h for the next 3 days. After medium changes, cells were starved for 16 h in RPMI 1640 containing 0.5% FBS to reduce background kinase activity.

The human proximal tubule epithelial cell line HK-2 and human T84 colonic carcinoma cells were maintained in Dulbecco’s Modified Eagle Medium/Nutrient Mixture F-12 (DMEM/F12; Corning, Thermo Fisher Scientific) media containing 10% FBS and supplemented with 5.0 μg/ml streptomycin and 5.0 U/ml penicillin at 37°C with 5% CO_2_ in a humidified incubator. HK-2 cells (2.0 × 10^5^ cells/well) and T84 cells (1.0 × 10^6^ cells/well) were seeded in 12-well plates, washed once with DPBS, and treated with 1.0, 5.0, or 10.0 ng/mL Stx1a or Stx2a for various time points in DMEM/F12 containing 0.5% FBS.

### Western Blot Analysis

Cells were lysed in a CETi lysis buffer (TransLab, Republic of Korea). Protein concentrations in the lysate were determined by Pierce BCA Protein Assay Kit (Thermo Fisher Scientific). 25–50 μg of protein was loaded in each lane of Bolt 4-12% Bis-Tris polyacrylamide gels (Invitrogen, USA). After electrophoresis, proteins were transferred onto polyvinylidene difluoride (PVDF) membranes and blocked for 1 h with 3% Bovine Serum Albumin (BSA) in TBST (20 mM Tris [pH 7.6], 137 mM NaCl, 0.1% Tween 20) to block nonspecific antibody binding. After 1 h of incubation, membranes were washed three times for 5 min each with TBST. After washing, primary antibodies were added before incubation at 4°C overnight. Membranes were subsequently washed three times before incubation with secondary antibodies labeled with horseradish peroxidase (HRP) for 2 h at room temperature in the dark. Bands were detected using the Immobilon Forte Western HRP substrate (Merck Millipore, USA) and an Odyssey Scanner (LI-COR, Germany).

### Knockdown siRNA and Overexpression

Specific SMARTpool siRNAs targeting Tristetraprolin and nontargeting negative-control siRNA were purchased from GE Healthcare Dharmacon, Inc. (USA). Macrophage-like THP-1 (1.0 × 10^6^ cells/well) or HK-2 (2.0 × 10^5^ cells/well) cells were transfected with each siRNA (100 pmol) in Opti-MEM using the *Trans*IT-X2 dynamic delivery system transfection reagent from TransIT-X2 (Mirus Bio, USA) according to the manufacturer’s instructions.

The human TTP expression vector was purchased from OriGene Technologies (USA). WT human TTP was used as a template for mutagenesis. A 993 bp fragment containing the phosphorylation site of mutated TTP was inserted into SgfI-MluI restricted pCMV6. Macrophage-like THP-1 (1.0 × 10^6^ cells/well) or HK-2 (2.0 × 10^5^ cells/well) cells were transfected with 1 μg WT or mutated vectors using lipofectamine 3000 (Invitrogen, Thermo Fisher Scientific) according to the manufacturer’s instructions.

After 48 h of transfection with either siRNAs or overexpression vectors, D-THP-1 or HK-2 cells were washed twice with DPBS and treated with Stx2a for 6, 12, and 24 h.

### Reverse Transcription-Quantitative Polymerase Chain Reaction (RT-qPCR)

Differentiated THP-1 or HK-2 cells were seeded in 12-well plates under the same conditions described above. Total RNA was extracted using NucleoSpin RNA Plus (Macherey-Nagel, Germany) according to the manufacturer’s instructions. cDNA was synthesized using reverse-transcriptase, and amplification was performed using a NanoHelix RT-qPCR kit (NanoHelix, Republic of Korea). The real-time PCR cycling conditions included a common amplification step with an initial cycle for cDNA synthesis at 50°C for 30 min, initial denaturation at 95°C for 12 min, 45 cycles of denaturation at 95°C for 20 s, and annealing and extensions at 60°C for 60 s. SYBR Green was used to quantify the results. Data were analyzed using LightCycler 96 System Software 1.1 (Roche Diagnostics GmbH, Germany). mRNA expression for all genes was normalized to the level of GAPDH. The primer sequences used for real-time PCR are listed in Table 1.

### Enzyme-Linked Immunosorbent Assays (ELISAs)

The concentrations of secreted soluble cytokines and chemokines in the culture medium were measured using ELISA (Invitrogen). Cellular supernatants were collected from D-THP-1 or HK-2 cells treated with Stx2 (10 ng/ml). Supernatants for TNF-α, IL-1β, MCP-1, MIP-1α, IL-6, IL-8, and IL-10 detection were assayed in triplicate wells in half-area microplates (Corning, USA) according to the manufacturer’s instructions. In brief, ELISA microplates were coated with 50 μl of capture antibody in 1x PBS (10 mM phosphate buffer, 2.7 mM KCl, 137 mM NaCl) overnight at 4°C. Wells were washed three times for 1 min with PBST (10 mM phosphate buffer, 2.7 mM KCl, 137 mM NaCl, and 0.05% Tween 20, [pH 7.4]) and blocked for 1 h at room temperature with 100 μl of 1x ELISA Diluent. Wells were then washed three times and incubated with 50 μl of each sample for 2 h at room temperature. Wells were washed again and incubated with 50 μl of detection antibody solution for 1 h at room temperature. Wells were washed an additional three times before incubation with 50 μl of streptavidin-HRP for 30 min at room temperature in the dark. Finally, wells were washed five times and incubated with 50 μl of substrate solution for 15 min at room temperature in the dark. After color development, 25 μl of 2 M H_2_SO_4_ was added to stop the reaction, and absorbance at 450 nm was measured using a SpectraMax 190 Microplate Reader (Molecular Devices, USA).

### Statistical Analysis

All data are expressed as mean ± SEM using GraphPad Prism version 5.00 (GraphPad Software, Inc., USA). Student’s *t*-test (for paired or unpaired samples) was used for statistical analysis. Values of *p* < 0.05 were considered statistically significant (*p < 0.05; **p < 0.01; ***p < 0.001).

## Results

### Stxs Induce the MK2-TTP Pathway Time- and Dose-Dependently

**Fig. 1:**
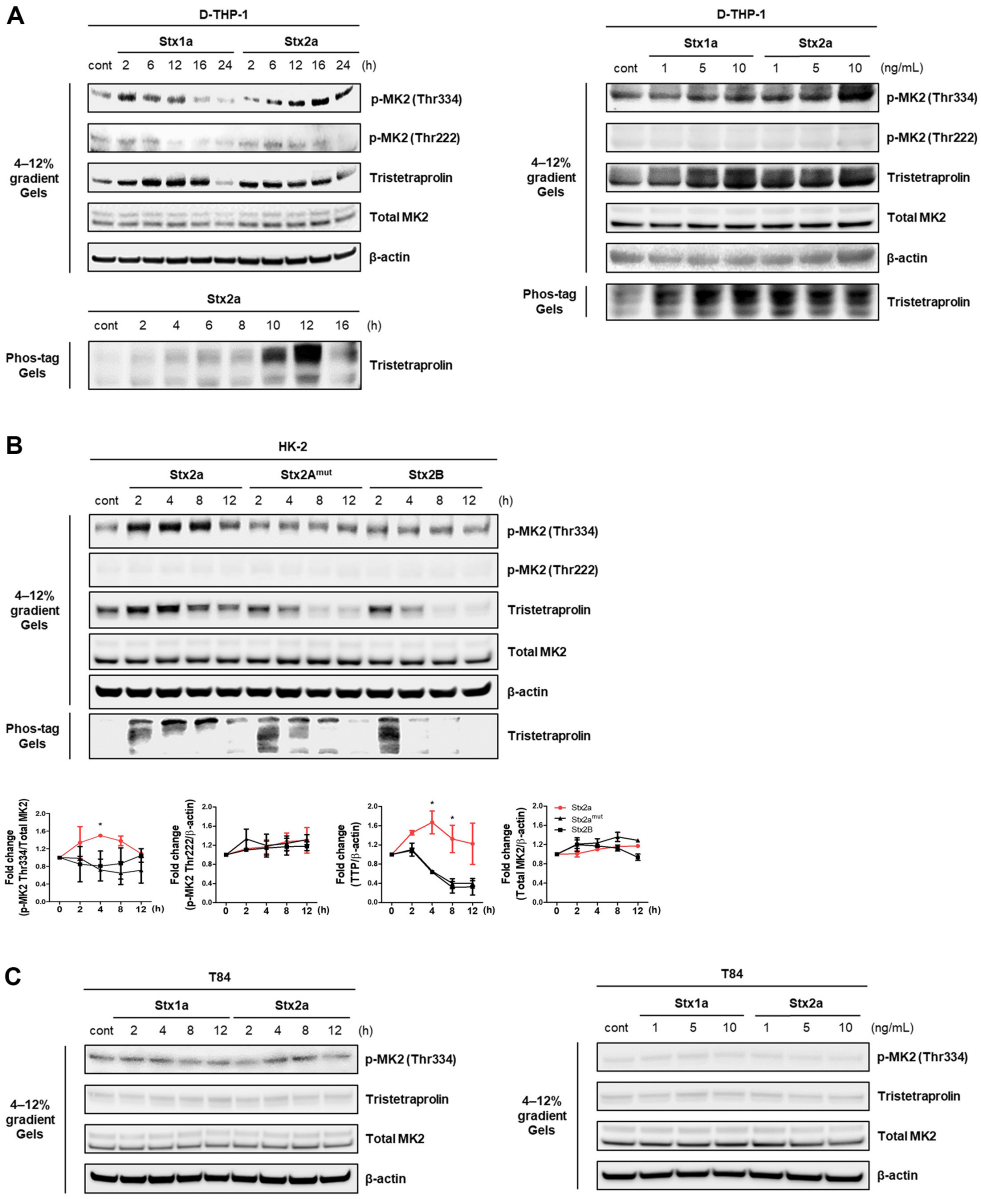
Stxs induce the MK2-TTP pathway in a time- and dose-dependent manner. (**A**) Gb_3_-positive differentiated THP-1 (D-THP-1) cells were stimulated with Stx1 and Stx2. D-THP-1 cells were treated with Stx1a (10 ng/ml) and Stx2a (10 ng/ ml) for the indicated time points (left panel). D-THP-1 cells were treated with various concentrations of Stx1a and Stx2a for 8 h. At the indicated times, cells were lysed, and the presence of phosphorylated MK2 and its downstream target TTP in cellular lysates were determined by western blotting using 4–12% gradient gels or Phos-tag gels as indicated (right panel). (**B**) MK2 and TTP phosphorylation in Stx2a-treated HK-2 cells. HK-2 cells were treated with various concentrations of Stx1a and Stx2a for 8 h. HK-2 cells were treated with Stx2a (10 ng/ml), Stx2a^mut^ (10 ng/ml), or Stx2B (10 ng/ml) for varying times (upper panel). Western blotting was used to measure the level of phosphorylated MK2 and TTP in lysates using 4–12% gradient gels or Phostag gels (lower panel). (C) The phosphorylation of MK2 and TTP does not change significantly in Gb_3_-negative T84 cells after Stxs exposure. Toxin receptor Gb_3_-deficient T84 cells were stimulated with Stx1 and Stx2. T84 cells were treated with Stx1a (10 ng/ml) and Stx2a (10 ng/ml) for the indicated times (left panel). T84 cells were treated with various concentrations of Stx1a and Stx2a for 8 h. Cell lysates were subjected to western blotting using antibodies against MK2 and TTP (right panel).

Previous studies showed that Stxs activate p38-MK2 signaling, promoting the activation of Hsp27 in Gb_3_-expressing cells [18]. To determine whether Stxs-induced phosphorylation of MK2 and TTP, we treated macrophage-like D-THP-1 cells with Stx1a and Stx2a for various time points (2–24 h) and doses (Stx1a 1–10 ng/ml; Stx2a 1–10 ng/ml) before analyzing cell lysates by western blotting. Phosphorylation of MK2 at Thr 334 and total expression of TTP increased in a time- and dose-dependent manner (Fig. 1A).

We then stimulated HK-2 cells with Stx1a and Stx2a, or Stx2a, Stx2a^mut^, and Stx2B, as above, and analyzed cell lysates by western blotting. As there is no commercially available antibody to measure phosphorylation of TTP, we ran reduced samples on Phos-tag gels, which slow the migration of phosphorylated proteins compared to their nonphosphorylated counterparts. Phosphorylation levels of MK2 at Thr 334 and TTP were dramatically increased between 4 and 12 h in both Stx1a- and Stx2a-treated HK-2 cells (Fig. S1, *left panel*). In agreement with Fig. S1A (*right panel*), the phosphorylation levels of MK2 at Thr334 and TTP increased dose-dependently after treatment with both Stx1a and Stx2a (1–10 ng/ml) (Fig. S1, *right panel*). Phosphorylation of MK2 Thr334 and total TTP expression were increased between 4 and 8 h in the presence of enzymatically active Stx2a, while enzymatically inactive toxin showed only limited activation of MK2-TTP pathways (Fig. 1B, upper panel). The phosphorylation of MK2 Thr334 by Stx2a was 2.0-fold greater than Stx2a^mut^ at 4 h post-treatment. The levels of total TTP induced by Stx2a increased by 2.5-fold at 4 h and 3.3-fold at 8 h when compared with Stx2a^mut^ at the same time points (Fig. 1B, lower panel).

The MK2 residues phosphorylated by p38 MAPK are Thr25, Thr222, Ser272, and Thr334 [30]. We observe that Stxs only induced phosphorylation of MK2 at Thr334, but not Thr222. There was no change in phosphorylation of MK2 Thr334 and TTP in Stxs receptor-negative T84 human colonic carcinoma cells (Fig. 1C).

### Decreased Expression of TTP Enhances Stx2a-Induced Inflammatory Responses

**Fig. 2:**
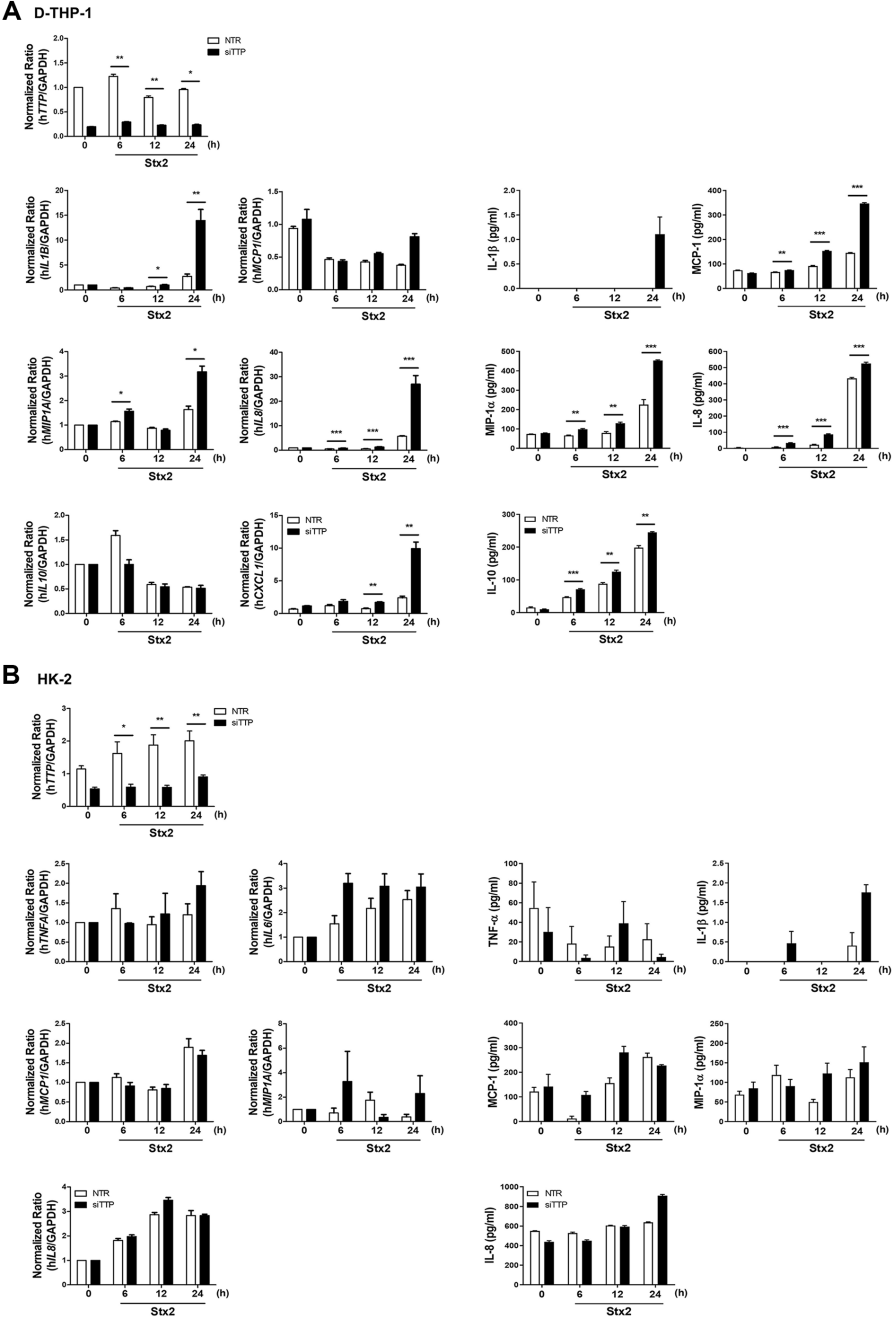
Decreased expression of TTP enhances Stx2a-induced pro-inflammatory responses. (**A**) Decreased expression of TTP enhances Stx2a-induced inflammatory responses in D-THP-1 cells. D-THP-1 cells were transfected with siRNA targeting TTP and nontargeting negative-control siRNA (NTR) for 48 h. After 48 h, transfected D-THP-1 cells were stimulated with Stx2a (10 ng/ml) for 6, 12, and 24 h, and total RNA was isolated. Knockdown of TTP was confirmed by transcription-quantitative PCR (RT-qPCR). Data were normalized against GAPDH. The transcriptional levels of cytokines and chemokines from TTP-deficient D-THP-1 cells were quantified by RT-qPCR. Data were normalized against GAPDH (left panel). Protein levels of the indicated cytokines/chemokines in culture supernatants from TTP-deficient D-THP-1 cells were measured by ELISA (right panel). Results are means ± SEM from two independent experiments, each performed in triplicate. (**B**) Decreased expression of TTP enhances Stx2a-induced inflammatory responses in HK-2 cells. HK-2 cells were transfected with siRNA targeting TTP and nontargeting negative-control siRNA (NTR) for 48 h before treatment with Stx2a (10 ng/ml) for 6, 12, and 24 h. Knockdown of TTP was confirmed by transcription-quantitative PCR (RT-qPCR). Data were normalized against GAPDH. Expression levels of *TNFA*, *IL6*, *MCP1*, *MIP1A*, and *IL8* mRNA from TTP-knockdown HK-2 were determined by RT-qPCR. Data were normalized against GAPDH (left panel). Culture supernatants from TTP-deficient HK-2 were collected, and TNF-α, IL-1β, MCP-1, MIP-1α, and IL-8 concentrations were measured by ELISA (right panel). Results are means ± SEM from two independent experiments, each performed in triplicate.

A previous study demonstrated that TTP-knockout increased IL-6 expression in BMDM from myeloid-specific TTP-deficient mice [31]. To determine if TTP expression altered proinflammatory signaling in Stx2a-intoxicated cells, we transfected D-THP-1 and HK-2 cells with siRNA targeting TTP or nontargeting siRNA (NTR) for 48 h, before stimulation with Stx2a. We confirmed that TTP expression in TTP-targeted siRNA-transfected D-THP-1 cells was reduced by 75% on average compared to NTR using RT-qPCR (Fig. 2A, upper panel). Cell-free culture supernatants were collected from D-THP-1 or HK-2 cells, and cytokine and chemokine concentrations were measured by ELISA. In TTP-deficient D-THP-1 cells, Stx2a induced greater *IL1B*, *MIP1A*, *IL8*, and *CXCL1* mRNA expression at 24 h post-treatment (Fig. 2A, left panel), and greater MCP-1, MIP-1α, IL-8, and IL-10 protein expression between 12 and 24 h than NTR controls (Fig. 2A, right panel).

We confirmed that TTP expression in HK-2 cells was reduced by 60% on average in siRNA-transfected cells compared to NTR using RT-qPCR (Fig. 2B, upper panel). In TTP-knockdown HK-2 cells, Stx2a induced increased *TNFA*, *IL6*, and *IL8* mRNA expression at 12 and 24 h post-treatment, and IL-1β, MIP-1α, IL-8, and MCP-1 protein expression at 12 and 24 h (Fig. 2B, lower panel). Our results demonstrate that TTP-deficient cells induce a more potent inflammatory response than NTR in Stx-stimulated Gb_3_-positive cells.

### Stx2a Inactivation of TTP Requires Phosphorylation at S60, S93, S90, and S323

**Fig. 3:**
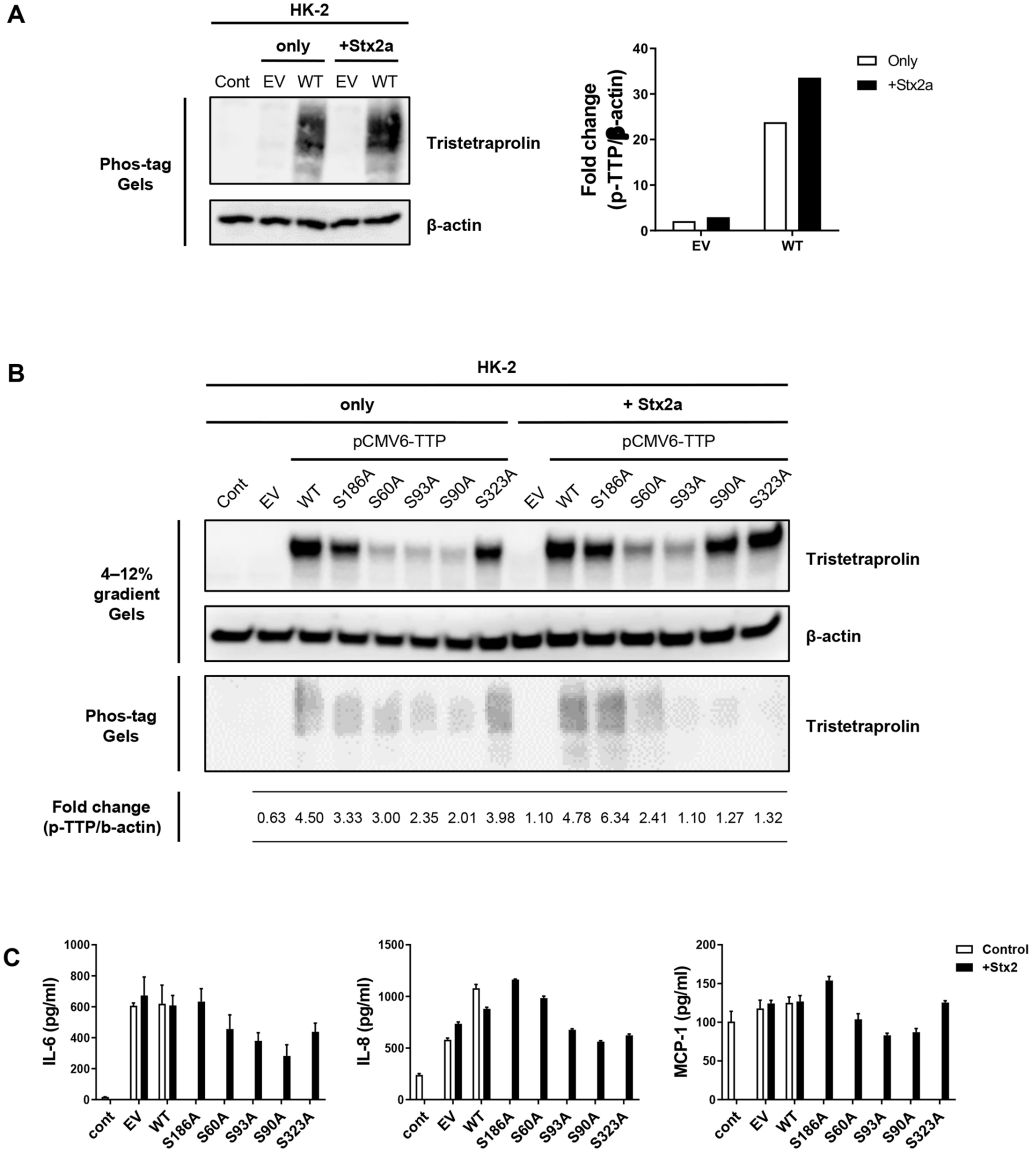
Stx2a-induced inactivation of TTP requires phosphorylation at key serine residues (S60, S93, S90, and S323) in HK-2 cells. (**A**) Stx2a induces TTP phosphorylation in HK-2 cells. HK-2 cells were transfected with either an empty vector (EV) or wild-type TTP (WT, pCMV6-TTP) for 48 hours. After transfection, cells were treated with Shiga toxin 2a (Stx2a, 10 ng/mL) for 8 h. The levels of phosphorylated TTP (p-TTP) were analyzed using Phos-tag acrylamide gels. Western blotting shows that Stx2a significantly increases TTP phosphorylation in WT-transfected cells compared to EV control. β-actin was used as the loading control. The bar graph (right) quantifies the fold change in phosphorylated TTP normalized to β-actin, demonstrating a significant increase in TTP phosphorylation in the presence of Stx2a in WT cells. (**B**) Phosphorylation site mutations reduce Stx2a-induced TTP phosphorylation. HK-2 cells were transfected with either EV, WT, or TTP mutants containing serine-to-alanine mutations at key phosphorylation sites (S186A, S60A, S93A, S90A, and S323A) for 48 h. After transfection, cells were treated with Stx2a (10 ng/ml) for 8 h. Total TTP protein was analyzed using a 4-12% gradient SDS-PAGE gel, and phosphorylated TTP was detected using Phos-tag acrylamide gels. β-actin was used as the loading control. The data show that phosphorylation of TTP was significantly reduced in the S60A, S93A, S90A, and S323A mutants, as indicated by the lower fold-change in p-TTP levels compared to WT, highlighting the critical role of these sites in Stx2a-mediated TTP phosphorylation. (**C**) Phosphorylation site mutations reduce Stx2a-induced proinflammatory cytokine production. The impact of TTP phosphorylation site mutations on the production of IL-6, IL-8, and MCP-1 was analyzed by ELISA. HK-2 cells were transfected as described in panel B and stimulated with Stx2a (10 ng/ml) for 8 h. Stx2a-induced cytokine production was significantly elevated in WT-transfected cells, whereas mutations at S60, S93, S90, and S323 notably reduced the secretion of IL- 6, IL-8, and MCP-1. This indicates that phosphorylation of TTP at these residues is essential for the Stx2a-induced proinflammatory response in HK-2 cells.

Phosphorylation of TTP occurs downstream of several signaling pathways, including ERK/MAPK, p38 MAPK, JNK, and PKB/AKT. Human TTP can be phosphorylated at Ser186, Ser60, Ser93, Ser90, and Ser323 [CST, PhosphoSitePlus v6.5.8]. We confirmed whether the phosphorylation of TTP is affected by Stx2a, and whether this is involved in Stx2a-induced inflammatory response regulation by mutating its five phosphorylation sites. Serine phosphosites were mutated to alanines and overexpressed using Lipofectamine 3000 in HK-2 cells. The overexpressed TTP was 1.4-fold phosphorylated after Stx treatment in HK-2 cells (Fig. 3A, left panel). Mutation of S60, S93, S90, and S323 to alanine prevented phosphorylation at these sites, resulting in decreased IL-6, IL-8 and MCP-1 concentrations in culture supernatants from Stx2a-stimulated HK-2 cells (Fig. 3B and 3C). These data suggest that S60, S93, S90, and S323 of TTP regulate phosphorylation by Stx2a, preventing TTP from reducing inflammation.

### Inhibiting MK2 Blocks the Phosphorylation of TTP and Reduces the Inflammatory Response by Stx2a

**Fig. 4:**
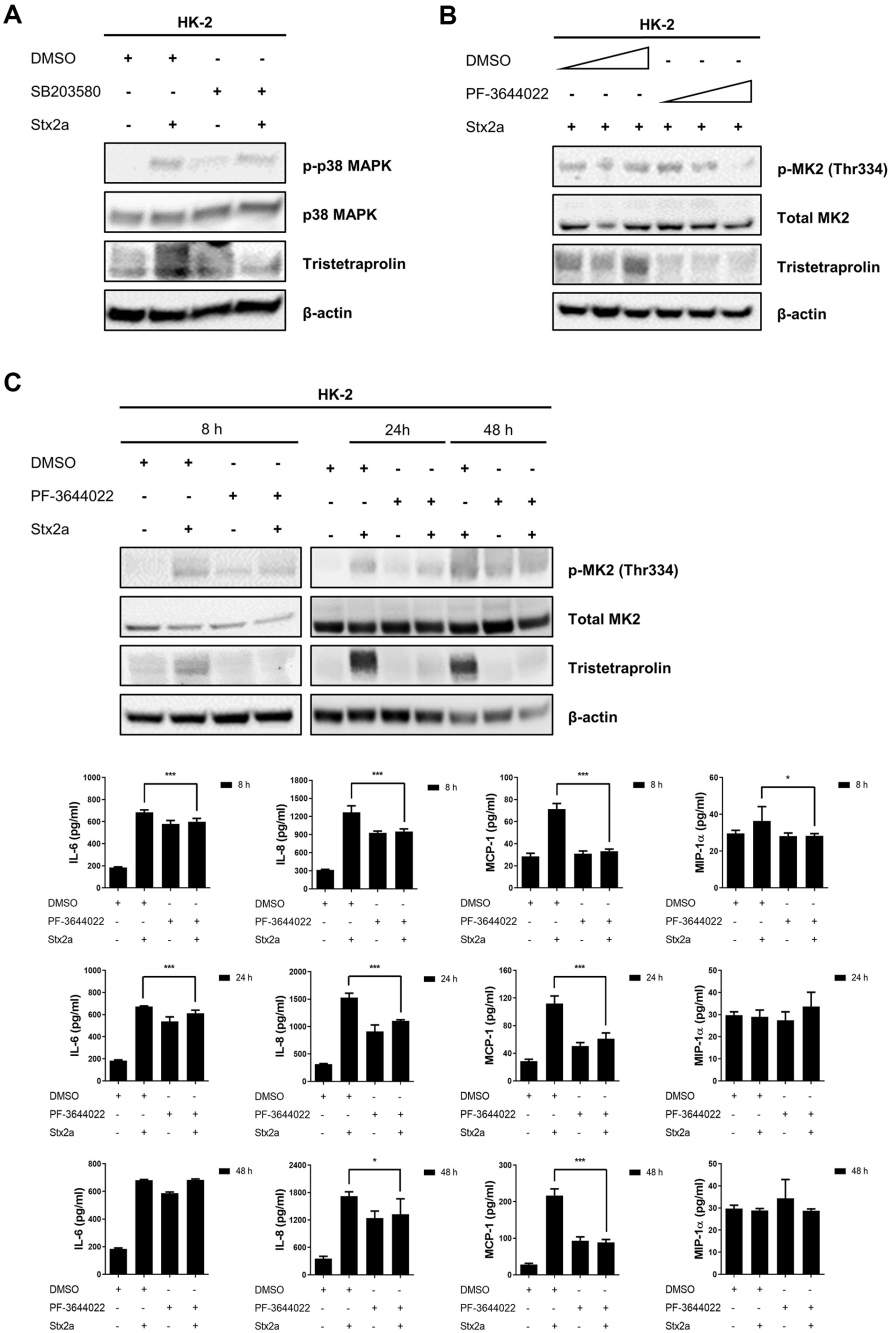
The MK2 inhibitor PF-3644022 suppresses the phosphorylation of TTP and reduces the inflammatory response induced by Shiga toxin 2a (Stx2a) in HK-2 cells. (**A**) p38 MAPK inhibition reduces Stx2ainduced phosphorylation of p38 and TTP expression. HK-2 cells were pretreated for 1 hour with DMSO or the p38 inhibitor SB203580 (10 μM) in DMEM/F12 medium containing 0.5% FBS, followed by treatment with Stx2a (10 ng/ml) for 8 h. Western blotting was performed to assess the phosphorylation levels of p38 MAPK (p-p38 MAPK), total p38 MAPK, and TTP expression. β-actin was used as a loading control. (**B**) MK2 inhibition reduces Stx2a-induced MK2 phosphorylation and TTP expression in a dose-dependent manner. HK-2 cells were pretreated for 1 h with DMSO or increasing concentrations of the MK2 inhibitor PF-3644022, followed by treatment with Stx2a (10 ng/ml) for 8 h. Western blot analysis was used to detect phosphorylated MK2 (p-MK2, Thr334), total MK2, and TTP expression. β-actin was used as a loading control. (**C**) Timedependent effects of MK2 inhibition on Stx2a-induced inflammatory cytokine production. HK-2 cells were pretreated for 1 hour with DMSO or the MK2 inhibitor PF-3644022 (10 μM), followed by treatment with Stx2a (10 ng/ml) for 8, 24, and 48 h. Western blotting was performed to detect p-MK2 (Thr334), total MK2, and TTP expression. In the lower panels, enzymelinked immunosorbent assays (ELISAs) were used to analyze the inhibitory effects of PF-3644022 on IL-6, IL-8, MCP-1, and MIP-1α secretion from HK-2 cells after Stx2a stimulation at the indicated time points (8, 24, and 48 h).

Regulation of TTP activity by p38 MAPK results in biphasic TNFα-induced *IL6* mRNA expression in human bronchial smooth muscle cells [32]. Conversely, dephosphorylation of TTP decreased the expression of IL-6 and IL-8 in A549 lung epithelial cells [33]. To investigate whether p38 MAPK is upstream of MK2 and thus inhibits TTP, we used the p38 MAPK inhibitor SB203580. A previously described MK2 inhibitor, ab146422, displayed no TTP inhibitory effect as it targeted Hsp25, a divergent substrate of MK2. HK-2 cells were preincubated with PF-3644022 for 1 h before treatment with Stx2a. Phosphorylation of p38 MAPK and TTP was reduced compared to cells treated with Stx2a in the absence of SB203580 (Fig. 4A). Further, TTP phosphorylation was also inhibited downstream of p38 MAPK by the MK2 inhibitor PF-3644022 in Stx2a-stimulated HK-2 (Fig. 4B). Inhibition of TTP using PF-3644022 reduced cytokine production, particularly IL-6, IL-8, MCP-1, and MIP-1α at 8 h post-treatment. Furthermore, inhibition of TTP phosphorylation at 24 and 48 h post-treatment decreased IL-6, IL-8, and MCP-1 secretion (Fig. 4C). These data suggest that MK2 inhibitors such as PF-3644022 reduce phosphorylation of TTP by Stx and inhibit Stx-induced inflammation.

## Discussion

Stxs exposure triggers an increase in various stress-activated kinase pathways, including p38 MAPK, JNK, ERK, and MK2, promoting the synthesis of inflammatory cytokines and chemokines. Hsp27 is phosphorylated by MK2 downstream of lipopolysaccharide (LPS) or Stxs, and the inflammatory response induced by Stxs could be reduced when p38 MAPK or MK2 was inhibited [18, 34]. MK2 readily phosphorylates substrates such as Hsp25, Hsp27, and TTP [19]. In particular, TTP is involved in the turnover of ARE-containing cytokine and chemokine mRNA.

We demonstrated that Stxs exposure in Gb_3_-expressing D-THP-1 cells promoted phosphorylation of TTP (Fig. 1A). We also found that TTP was predominantly phosphorylated in HK-2 cells treated with Stx2a in a time manner (Fig. 1B). Gb_3_-negative cells, however, showed no change in the phosphorylation state of TTP or the upstream kinase MK2 (Fig. 1C). TTP expression was shown to be induced by LPS, which subsequently blocked TNF-α production in BMDMs of WT mice [35].

We observed changes in the Stxs-induced inflammatory response in both cells depleted of TTP by siRNA transfection. TTP-deficient THP-1 or primary human macrophages increased *TNFA* and *IL1B* production after LPS exposure [33]. BMDMs from myeloid-specific TTP-knockout mice produced more *IL6* than did WT after LPS treatment [31]. Similarly, in our study, TTP-knockdown led to increased *IL1B*, *MCP1*, *MIP1A*, *IL8*, *IL10* mRNA, and protein expression in Stx2a-stimulated D-THP-1 cells (Fig. 2A). In addition, TTP-knockdown in HK-2 cells led to increased *TNFA*, *IL1B*, *IL6*, *MCP1*, *MIP1A*, *IL8* mRNA, and protein expression by Stx2a (Fig. 2B). These data suggest that nonphosphorylated TTP is the active state of this protein, resulting in negative regulation of cytokine production, which is abrogated by Stxs-induced phosphorylation.

Overexpression of non-phosphorylated S316A TTP mutant enhanced TTP activity and decreased TNF-α mRNA [35]. We mutated the phosphorylation sites of human TTP from serine to alanine to observe the change in phosphorylation levels. TTP mutations S60A, S90A, and S323A decreased phosphorylation and the subsequent Stx2a-induced production of cytokines, including IL-6, IL-8, and MCP-1 (Fig. 3). These results suggest that specific TTP phosphorylation sites targeted by Stx2a prevented degradation of cytokines and chemokines mRNA. Total protein western blots indicate that MK2-mediated TTP phosphorylation at S60, S93, S90, and S323 regulates protein turnover during Stx2a-induced inflammatory responses.

Stx2a-mediated TTP phosphorylation is dependent on the p38 MAPK-MK2 pathway. Chemical compound PF-3644022 inhibits phosphorylation of MK2 and Hsp27, and blocks LPS-stimulated TNF-α production [37, 38]. PF-3644022 also suppresses the phosphorylation of TTP by MK2, preventing inactivation of TTP. We used ELISA to show that IL-6, IL-8, MCP-1, and MIP-1α production was decreased in PF-3644022-treated HK-2 cells, suggesting that activated TTP decreases the inflammatory response to Stx2a (Fig. 4).

**Fig. 5:**
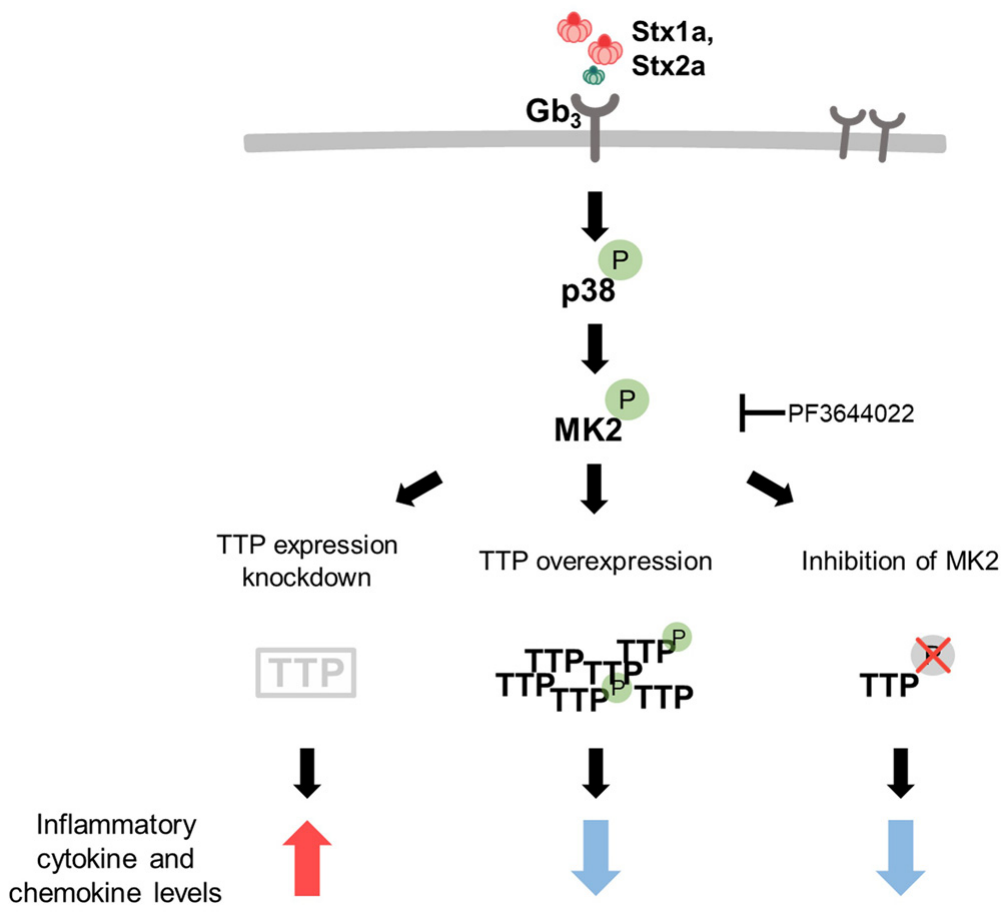
The MK2-TTP signaling pathway modulates Shiga toxin (Stx)-induced inflammatory responses in toxin-sensitive cells. Schematic model depicting the regulation of Stx1a/Stx2a-induced inflammatory responses via the MK2-TTP signaling pathway. EHEC Stx1a and Stx2a bind to the Gb_3_ receptor on toxin-sensitive cells, activating the p38 MAPK signaling pathway, which in turn phosphorylates MK2. Phosphorylated MK2 then phosphorylates TTP, leading to its inactivation. This inactivation of TTP results in the stabilization of proinflammatory cytokine mRNAs, thereby increasing the levels of inflammatory cytokines and chemokines. This model integrates the effects of TTP phosphorylation and MK2 inhibition (as shown in the data) on the regulation of IL-6, IL-8, and MCP-1 production in response to Shiga toxins in HK-2 cells, as described in the previous figures.

Although the current study was conducted only in vitro, there is a possibility of reducing the inflammatory response to Stx by inhibiting phosphorylation of TTP in vivo in humans. Our results suggest that TTP may play an important regulatory role in the Stx-induced inflammatory response (Fig. 5). Further studies are needed to confirm the role of TTP in reducing inflammatory responses in vivo using inhibitors.

## Conclusion

In this study, we have demonstrated that Shiga toxins (Stxs) from enterohaemorrhagic *E. coli* activate the MK2-TTP signaling pathway, leading to the production of inflammatory cytokines in toxin-sensitive cells. We observed that phosphorylation of MK2 at Thr334 and TTP is critical for the Stx2a-mediated inflammatory response, as these modifications stabilize cytokine mRNAs, including *IL6*, *IL8*, and *MCP1*. Notably, mutations at key phosphorylation sites of TTP (S60, S90, S93, and S323) significantly attenuated cytokine production, emphasizing the importance of TTP phosphorylation in the regulation of inflammation. Furthermore, inhibition of MK2 using PF-3644022 blocked the phosphorylation of TTP and decreased cytokine production, suggesting a potential therapeutic approach to mitigate the inflammatory response induced by Stxs. Overall, our findings highlight a novel mechanistic role of the MK2-TTP axis in Stx-mediated pathogenesis, offering valuable insights for the development of targeted therapies in Shiga toxin-associated diseases, including hemolytic uremic syndrome.

## Supplemental Materials

Supplementary data for this paper are available on-line only at http://jmb.or.kr.



## Figures and Tables

**Table 1:** Sequence of RT-qPCR primers.

Gene	Type	Primer sequence 5’- 3’	Ref.
*TTP*	Forward	TGGGATCCGACCCTGATGAA	This study
	Reverse	TCGAAGACGGGAGAGTCAGA	
*TNFA*	Forward	AGCCCATGTTGTAGCAAACC	[24]
	Reverse	TCTCAGCTCCACGCCATT	
*IL1B*	Forward	GGACAGGATATGGAGCAACAA	This study
	Reverse	CCCAAGGCCACAGGTATTT	
*IL6*	Forward	GATGAGTACAAAAGTCCTGATCCA	[25]
	Reverse	CTGCAGCCACTGGTTCTGT	
*IL8*	Forward	GGTATCCAAGAATCAGTGAAGA	[26]
	Reverse	CTACAACAGACCCACACAATA	
*IL10*	Forward	GCCTAACATGCTTCGAGATC	[27]
	Reverse	TGATGTCTGGGTCTTGGTTC	
*MCP1*	Forward	GTCTCTGCCGCCCTTCTGTG	[28]
	Reverse	AGGTGACTGGGGCATTGATTG	
*MIP1A*	Forward	CTCTCTGCAACCAGTTCTCTG	This study
	Reverse	CTCGTCTCAAAGTACTCAGCTATG	
*CXCL1*	Forward	CCCAAGAACATCCAAAGTGTGA	This study
	Reverse	CAAGCTTTCCGCCCATTCT	
*GAPDH*	Forward	ATCTTCAAGCCATCCTGTGTGC	[29]
	Reverse	TGCGCTTGTCACATTTTTCTTG	
