# Supplementary material for: Shiga Toxins Produced by Enterohaemorrhagic Escherichia coli Induce Inflammation in Toxin-Sensitive Cells through the p38 MAPK/MK2/Tristetraprolin Signaling Pathway
Source: J Microbiol Biotechnol. 2024 Nov 22;34(12):2439–49. doi: 10.4014/jmb.2410.10016 (PMC11729694; doi:10.4014/jmb.2410.10016)
Supplement: Supplementary file 1 [file jmb-34-12-2439-supple.pdf]

J. Microbiol. Biotechnol. 2024. 34(12): 2439–2449

https://doi.org/10.4014/jmb.2410.10016

Supplementary Figure 1.

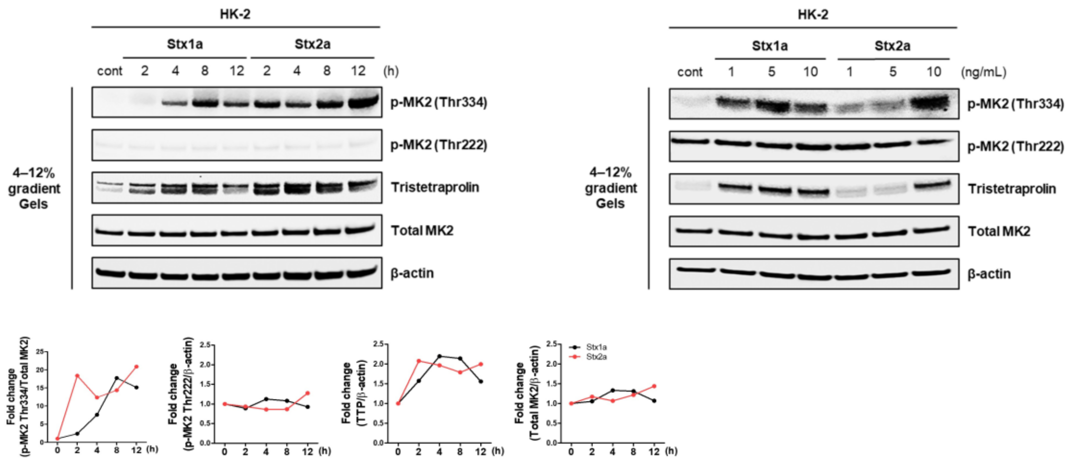

Supplementary Fig. S1. Time- and dose-dependent effects of Stx1a and Stx2a on MK2 phosphorylation and Tristetraprolin expression in HK-2 cells. (*left panel*) HK-2 cells were treated with Stx1a or Stx2a (10 ng/mL) for the indicated time points (2, 4, 8, and 12 hours). Western blotting was performed to assess the phosphorylation levels of MK2 at two key phosphorylation sites, Thr334 (p-MK2 Thr334) and Thr222 (p-MK2 Thr222). The expression levels of TTP and Total MK2 were also measured. β-actin was used as a loading control. (*right panel*) HK-2 cells were treated with increasing doses of Stx1a or Stx2a (1, 5, and 10 ng/mL) for 8 hours. Western blotting was performed to detect p-MK2 Thr334, p-MK2 Thr222, Tristetraprolin, and Total MK2. β-actin was used as a loading control.
